# Information resource preferences by general pediatricians in office settings: a qualitative study

**DOI:** 10.1186/1472-6947-5-34

**Published:** 2005-10-14

**Authors:** George R Kim, Edward L Bartlett, Harold P Lehmann

**Affiliations:** 1 Division of Health Sciences Informatics, Johns Hopkins University School of Medicine, Baltimore, Maryland, USA; 2 Department of Pediatrics, Johns Hopkins University School of Medicine, Baltimore, Maryland, USA

## Abstract

**Background:**

Information needs and resource preferences of office-based general pediatricians have not been well characterized.

**Methods:**

Data collected from a sample of twenty office-based urban/suburban general pediatricians consisted of: (a) a demographic survey about participants' practice and computer use, (b) semi-structured interviews on their use of different types of information resources and (c) semi-structured interviews on perceptions of information needs and resource preferences in response to clinical vignettes representing cases in Genetics and Infectious Diseases. Content analysis of interviews provided participants' perceived use of resources and their perceived questions and preferred resources in response to vignettes.

**Results:**

Participants' average time in practice was 15.4 years (2–28 years). All had in-office online access.

Participants identified specialist/generalist colleagues, general/specialty pediatric texts, drug formularies, federal government/professional organization Websites and medical portals (when available) as preferred information sources. They did not identify decision-making texts, evidence-based reviews, journal abstracts, medical librarians or consumer health information for routine office use.

In response to clinical vignettes in Genetics and Infectious Diseases, participants identified Question Types about *patient-specific *(diagnosis, history and findings) and *general medical *(diagnostic, therapeutic and referral guidelines) information. They identified specialists and specialty textbooks, history and physical examination, colleagues and general pediatric textbooks, and federal and professional organizational Websites as information sources. Participants with access to portals identified them as information resources in lieu of texts.

For Genetics vignettes, participants identified questions about prenatal history, disease etiology and treatment guidelines. For Genetics vignettes, they identified patient history, specialists, general pediatric texts, Web search engines and colleagues as information sources. For Infectious Diseases (ID) vignettes, participants identified questions about patients' clinical status at presentation and questions about disease classification, diagnosis/therapy/referral guidelines and sources of patient education. For ID vignettes, they identified history, laboratory results, colleagues, specialists and personal experience as information sources.

**Conclusion:**

Content analysis of office-based general pediatricians' responses to clinical vignettes provided a qualitative description of their perceptions of information needs and preferences for information resource for cases in Genetics and Infectious Diseases. This approach may provide complementary information for discovering practitioner's information needs and resource preferences in different contexts.

## Background

Information behaviors of pediatricians have been examined in different clinical environments: in general patient care at an academic medical center [1,2], in neonatal intensive care [3] and in hospital on-call care [4], and as part of a subset of practitioners caring for patients in a broader age range [5]. Collectively, these studies demonstrate distinctions in information resource preferences by pediatric practitioners in different clinical environments. A systematic review of information seeking behaviors of physicians in general [6] found wide variation of information resource preferences, distributed among colleagues, text sources and electronic databases, from which the authors inferred a need for further categorization of information needs and resources.

Published studies on pediatricians' information use have focused on hospital-based practitioners and house staff, although most pediatricians practice in office settings, away from medical libraries, librarians and hospital-based specialists [7]. Office-based practitioners have perspectives distinct from hospital-based practitioners that may influence their information needs and resource preferences. Among these perspectives may be practical constraints, such as the need to transport (rather than manage) unstable patients or the cost and space needed to maintain onsite repositories of information.

In contrast to these constraints has been the increasing adoption of information and communication technologies (ICT) into practice [8]. In addition to patient management tools such as electronic medical records (EMRs), electronic prescribing and secure messaging, pediatric practitioners have a growing number of traditional and new electronic knowledge resources available by subscription and for free, from medical center libraries [9,10], government agencies [11], professional organizations [12] and medical publishers. With increasing availability of telemedicine services [13] and marketing of inpatient specialty services as "product" [14], the information environment of the academic medical center is expanding to include community physicians' offices.

Domain competencies for US pediatricians have traditionally been defined by formal training curricula and board certification [15], but information management competencies linked to professional certification maintenance for continuous professional development (CPD) [16] and evidence-based practice improvement [17] are evolving. The day-to-day management of knowledge and incorporation of evidence into care have not been well defined [18] and are part of an informal practitioner curriculum [19], based on personal experience, availability and reputations of resources and local organizational cultures [20] that may influence what is and is not used.

This pilot study explores information needs and resource preferences of (a) a characterized group of practitioners (general pediatricians) within (b) a characterized clinical workflow (office-based practice) for (c) problems in characterized domains (genetics and infectious diseases) using short clinical vignettes. Vignettes [21] have been used to teach and evaluate problem-solving skills and to elicit practitioners' information needs [22]. Their advantages include cost effectiveness and case-mix control for new or rare conditions. Genetics and Infectious Diseases were chosen as two representative domains in which pediatricians receive formal training and clinical experience.

In this study, we explore several questions:

• Can vignettes be used to elicit information needs and resource preferences from a group of practitioners with common attributes, clinical environments and patient problems?

• What are information needs and resource preferences of office-based general pediatricians, and are there commonalities with pediatricians in other settings?

## Methods

### Design

We conducted semi-structured interviews of twenty office-based general pediatricians to elicit their: (a) demographic and computer use data, (b) verbalized uses of specified information resources and (c) "think aloud" perceptions of questions and identification of information resource preferences in response to short vignettes representing clinical cases in genetics and infectious diseases. This study was approved by the appropriate institutional review board.

### Setting

Participants were interviewed individually by telephone by one of the authors (GRK).

### Subjects

A sample of twenty general pediatricians was recruited from urban/suburban office-based practices responding to general calls for subjects through two professional pediatric group newsletters (Winter-Spring 2004), through hospital pediatric department and joint community practice affiliations and through direct telephone/e-mail solicitation. Participants were enrolled by telephone.

### Vignette creation

Sixteen short vignettes [See Additional file 1] representing hypothetical patients with problems in Genetics (8) or Infectious Diseases (8) were created to provide a variety of cases reflecting different combinations of familiarity, acuity and information problem (diagnosis or therapy) to general pediatricians. Domain experts in general pediatrics reviewed all vignettes (GRK, ELB, HPL) for presentation.

### Data collection

The vignettes were randomized into non-repeating sequential groups of four vignettes and presented to participants by telephone in a standardized fashion. Participants' verbal responses were recorded and transcribed immediately post-interview for content analysis. Interviews lasted approximately 30 minutes each. One participant was replaced after it was discovered post-interview that the audio recorder had malfunctioned.

For each of the twenty participants, after a standard introduction to the study and consent, data was collected in a continuous three-part fashion:

1. In response to each of four sequential, randomized vignettes, the participant was asked to state perceptions of the questions presented by the vignette and how s/he would approach the problems in a "think-aloud" fashion [23], indicating information resources that s/he would consult in managing or approach the vignette. For each information resource indicated, the participant was asked what s/he would do if the resource consulted did not have the answer, until the problem was "resolved".

2. In response to a list of medical and pediatric information resources, the participant was asked to state the types of questions, problems or information needs for which s/he would use each resource type.

3. Information about demographics (practice type, years in practice, computer and Internet availability) was elicited.

### Data analysis

Demographic information was tabulated for descriptive analysis [see Results: Demographics].

Indicated use of specific information resource types and their relative frequencies of indicated use (using the participant-resource pair as the unit of analysis) were tabulated for descriptive analysis [see Additional file 2 and Results: Information resources survey].

Transcribed responses to vignettes were coded consecutively according to the constant comparison method using open coding [24] to identify questions and information resources using the participant-vignette pair as the unit of analysis. Content analysis of interview transcripts was performed (GRK, HPL) using a combination of open-source software (AnSWR [25] from the Centers for Disease Control and Prevention) and desktop relational database/spreadsheet tools. Discrepancies were resolved through discussion (GRK, ELB).

Codes representing questions perceived from vignettes were sorted into nine categories forming two broad categories of Question Types about patient-specific and general medical information [see Additional file 3]. Question Types involving general medical knowledge were mapped to the closest entity from a published taxonomy of Generic Questions [26]. Codes representing information resource preferences were sorted into Information Resource Types describing patient-specific and general medical information sources [see Additional file 4]. Relative frequencies of perceived Question Types [see Additional file 5] and identified Information Resource preferences for all vignettes and for vignettes according to domain (genetics or infectious diseases) [see Additional file 6] were tabulated [see Results: Responses to vignettes].

### Validation

Validation of the results was by member-checking. A summary of the results [see Results] was presented to all participants with a 90% return. All returns concurred with the summary.

## Results

### Demographics

#### Participants and work environments

Twenty pediatricians (11 male, 9 female) in urban/suburban pediatric practice participated. Average time in practice was 15.4 yrs (range 2–28 yrs, median 17 years (male mean 17.5 yrs, median 19 yrs; female mean 12.7 yrs, median 16 yrs; t-test NS)). Twelve participants classified their practices as private practice, 4 as managed care and 4 as free clinic/federally qualified health center. All 20 classified their clinical work as general pediatrics, with 7 adding adolescent medicine, 2 adding adult medicine and 1 adding developmental pediatrics. Thirteen worked in private offices, 4 in multiple specialty settings, 2 in multi-site clinics and 1 in an expanded urgent care (with limited well child care).

#### Information resources and computer availability

All participants shared office computers with Internet access in clinical areas. All had billing functions performed by separate computer systems. Twelve had access to a private networked computer, 10 used handheld computers, 6 had wireless access, 4 had mobile computers and 4 used electronic prescribing. Three used an EMR, 4 had access to an EMR that provided read-only capability. Three used computers in consultation rooms. Two had computers for other projects (research and patient education). Twelve had the capability to communicate with their patients by e-mail, but only 2 did so more than very rarely.

### Information resources survey

#### Frequently and rarely identified resources

The most frequently identified resources were specialists, generalist colleagues and general pediatric texts, followed by drug formularies and specialty textbooks. Professional organization and federal health Websites (in particular the American Academy of Pediatrics and the Centers for Disease Control and Prevention) were identified as sources of guidelines and other information. When commercial medical portals were available (usually through institutional affiliation), they were frequently identified as a consulted information resource.

Resources rarely or not identified as sources of information in the office included: algorithmic/flow-chart decision-making texts, evidence-based medical reviews, medical journal abstracts, medical librarians or consumer health information. Several participants identified evidence-based medicine resources for specific problems or for self-education.

#### Identified uses of resources

Pediatric handbooks (such as the Harriet Lane Handbook [27]) were identified for drug dosing, cardiology information (electrocardiogram interpretation, blood pressure/pulse normal values) and disease-specific protocols. General pediatric texts were identified as first resources for resolving differential diagnosis and management questions, for general review and for self-education. Specialty texts were identified for domain-specific questions, with infectious disease (AAP Red Book [28]), genetics (Smith's Recognizable Patterns of Human Malformation [29]) and dermatology resources identified most frequently. Formularies were identified for questions on drug dosages, adverse reactions, packaging information, and for general information about drugs with which a participant had little or no experience. Guidelines and organizational policy statements, in particular those from the American Academy of Pediatrics [30], were identified for questions on disease management, for continuing education and for reference in policy-making.

Generalist colleagues were identified for confirmation of findings and for discussion of diagnostic and management decisions. Specialists were identified as information resources in anticipation of referral. Contact with generalist colleagues was usually face-to-face (in the office) while contact with specialists was via telephone and with a person or institution with which the participant had prior interaction or contact through an established referral network.

The CDC Travel Health Website [31] was the most frequently identified online resource for current immunization and travel health information. The CDC Website [11] was also identified for general immunization information. The American Academy of Pediatrics general Website [12] was identified for policy statements, current professional news and consumer health information. Commercial medical portals, when available, were identified in lieu of print textbooks.

### Responses to vignettes

#### Frequently perceived question types

##### Patient-specific question types

Collectively, patient-specific Question Types regarding details about history, findings (symptoms, signs, test results), diagnosis, and treatment plans were the most frequently perceived, followed by Question Types regarding current patient state (stability, general appearance/condition), social support and patient/family understanding of a condition. Participants also perceived pragmatic questions about legal and insurance issues regarding minors in non-custodial care.

Patient-specific questions about maternal, prenatal and birth history were perceived more frequently in response to Genetics vignettes and questions about patients' appearance/condition were perceived more frequently in Infectious Disease vignettes.

##### General medical question types

Collectively, general medical Question Types regarding etiology, and treatment/diagnosis guidelines for a condition were the most frequently perceived.

General medical questions about disease etiology occurred more frequently in response to Genetics vignettes and questions about diagnosis/referral criteria and disease classification were perceived more frequently in Infectious Disease vignettes.

Most general medical Question Types posed by participants had correspondences to Generic Medical Questions in the taxonomy developed by Ely et al [26], however, there were two for which there were no explicit matches:

• "What are the classifications of a disease?" (to decide on an approach to management):

• "...what type of sickle cell [the patient] has, if he has SS [a type of sickle cell disease] or one of the more favorable ones..."

• "...what type of school failure, is it in one area, speech?"

• "What is available treatment?" (to locate new and uncommon resources):

• "...what treatments are available, what's experimental? If it exists, where is it provided? Would [the patient] qualify for participation in a study?"

• "What is the latest therapy available for patients with a disease X?"

#### Frequently identified information resources

Collectively, specialists were the most frequently identified Information Resource Type. They were also the most frequently identified last resource when an answer could not otherwise be found.

##### Patient-specific resources

The most frequently identified patient-specific resources were patient history and examination, followed records from and communication with previous providers and institutions (inpatient hospital or nursery), which were similar for both Genetics and Infectious Diseases vignettes.

##### General medical resources

After specialists, the most frequently identified general medical resources were specialty and general pediatric texts and colleagues (pediatric generalists), although participants with access to medical portals indicated them as resources in lieu of texts. Participants also identified Web search engines, guidelines from professional organizations (American Academy of Pediatrics) and local institutions (hospitals, social services, health departments, schools).

For Genetics vignettes, genetic specialists, dysmorphology texts/atlases and genetic databases (Online Mendelian Inheritance in Man (OMIM) [32]) were more frequently identified than for Infectious Diseases vignettes, for which specialists in infectious disease and other domains, infectious disease texts and guidelines (in particular the AAP Red Book), federal and local government resources (CDC Website and local health departments, schools and social services) and their own experience and training were more frequently identified.

## Discussion

### Emerging model

The information process of physicians in patient care has been described as iterations of inquiries/interventions (history, examination, diagnostic testing, treatment) and responses to updates of a patient's clinical status and/or a practitioner's information state [33]. Parallel to this process is the process by which decisions are made about further inquiries/interventions, for which the practitioner may need to consult external resources [34].

Practitioners anticipate information needs by organizing and maintaining "personal information collections, defined as subsets of... information...that individuals [build] conceptually and physically over time" [35], including "sources and channels...that can be located again easily." These preferences follow a principle of "least effort" [36], in which selection of resources (tools) are optimized to meet needs (jobs) in terms of the effort and cost to maintain and use them.

Characterization of such collections and their use by practitioners may be helpful on several fronts. First, it can help plan placement of resources to promote their effective use. It has recently been suggested that the way to promote evidence-based medical practice is to promote information management [37]. Second, it may help to determine practitioner training needs (knowing what questions to ask and what resources to use) in problems of new awareness. A recent systematic review [38] suggests that the longer physicians are practice, the greater their need for quality improvement interventions. Third, it may help practitioners develop and maintain situational awareness (SA) of information as it relates to patient care, practice improvement and professional development. SA of clinical information has been described within an intensive care unit (ICU) [39], but not within physician offices. Lastly, it may help determine factors that influence practitioners to use specific resource types.

### Participants' information needs

In response to vignettes, patient-specific questions focused on patient history, examination and management. General medical questions focused on etiology, diagnostic/therapeutic management and guidelines. For Genetics vignettes, participants perceived questions about disease etiology more frequently than for Infectious Disease vignettes, for which they perceived questions about diagnostic and referral guidelines, disease classification and sources of patient education materials.

In comparison to an analysis of patient care questions asked at an academic medical center (AMC), the office-based (OB) participants perceived a higher relative frequency of questions about diagnosis and no questions about drugs. Reasons to explain this may include: 1) the OB group used hypothetical patients whereas the AMC-derived group used actual patients, 2) the OB group used only outpatient settings (where patient diagnoses are usually not known prior to the encounter) whereas more than half (58.8%) of the AMC-derived questions were generated from inpatient settings (where patients' admitting diagnoses are known), 3) the OB group used hypothetical encounters (where details of therapy may not be specified), whereas the AMC-derived group used actual patient cases and 4) the vignettes elicited questions about hypothetical cases in genetics and infectious diseases, whereas the AMC-derived questions came from patients from a number of different domains.

### Question taxonomies

As noted in the AMC study of pediatricians' questions, the participants' questions fell within a few Generic Questions from the taxonomy derived from ethnographic observation of family practitioners. Interestingly, participants perceived two Question Types that did not have specific matches within the taxonomy: questions about disease classifications and about sources of the best and latest care for a disease. One suggestion that has been made is that pediatricians may have different information needs from family practitioners [1].

To express information needs of a group of practitioners, a question taxonomy should: a) match their *perspectives *(Example: parenteral nutrition and intravenous fluids are largely inpatient, not outpatient objects), the *inventory *(of objects/relationships) relevant to that perspective (Example: nutrition and immunizations may be therapy, but are not drugs) and the *granularity *(of knowledge) needed (Example: parenteral nutrition, infant formula and breastfeeding are distinct and important forms of nutrition) to express concepts and information needs easily and clearly [40]. The Generic Question taxonomy, while important, may not sufficiently express the practical information needs of pediatricians.

### Participants' information resources

Within the context of Genetics and Infectious Diseases (as represented in these vignettes), participants identified a relatively small, but common set of defined resource types (general and specialty pediatric texts, generalist colleagues). They named a few specific, trusted online resources, although some were comfortable with "open" Web searching. They identified trusted and established sources of specialty care (frequently through a medical center), reachable by phone.

In comparison to AMC pediatricians, office-based general pediatricians have similar resource preferences, but also identified community resources, including the patient's family and social environment (school, etc.). Participants identified specific Websites (CDC, AAP) and medical portals when available (through academic affiliations) as trusted sources. In contrast to AMC-defined roles of "faculty" and "resident," participants identified in-office colleagues as informal consultants and specialists (by phone) as formal consultants. Specialists were frequently identified as "last" resource if an answer could not otherwise be found.

### The use of vignettes

The described use of vignettes can generate a description of information resource use, but it does not provide information on what users actually do. It also does not provide information on barriers to pursuing answers to questions. It is not intended to supplant other methods of discovering information needs (such as surveys, interviews and ethnographic observations), but as a way of exploring information needs and resource preferences in a flexible and low-cost fashion. It may be useful in "fine-tuning" exploration of information needs and resource preferences:

• Vignettes about rare or new conditions may be used to identify practitioners' unrecognized information needs and/or to guide the development and deployment of new information resources to fulfill needs

• Vignettes about new morbidities may be used to identify the unrecognized practitioner information needs and/or to guide the development of practitioner education and awareness programs

In addition to the qualitative frequency analyses described in this paper, exploratory factor analysis of the results may be used to discover categories of resources that may help determine their selection by practitioners in different circumstances (using a Hierarchy of Abstraction Model [41]).

## Conclusion

Using content analysis of semi-structured responses to short clinical vignettes in the domains of genetics and infectious diseases, we have explored information needs and resource preferences of office-based general pediatricians. This approach yielded descriptive information that demonstrates commonalities and distinctions with other studies on the information needs of general pediatricians.

## Competing interests

The author(s) declare that they have no competing interests.

## Authors' contributions

GRK participated in the design of the study and obtaining IRB approval, developed the vignettes and questionnaire, organized agreement on attribute assignments, recruited participants, interviewed the participants, coded transcripts (simple, axial and selective), and drafted the manuscript. ELB participated in the design of the study and obtaining IRB approval, tested the vignettes and reviewed the questionnaire and reviewed the manuscript. HPL participated in the design of the study and obtaining IRB approval, helped in the development of vignettes and questionnaire, participated in agreement on attribute assignments, participated in coding of transcripts and reviewed the manuscript. All authors read and approved the final manuscript.

## Pre-publication history

The pre-publication history for this paper can be accessed here:



## Supplementary Material

Additional file 1 **Vignettes used in semi-structured interviews **Sixteen vignettes listed by domain (Genetics (8), Infectious Diseases(8)) Click here for file

Additional file 2 **Information resource survey **Relative frequency of resource use identified by participants. Cited uses of specific resources Click here for file

Additional file 3 **Question types perceived in vignettes **Patient-specific and general medical question types perceived by participants in response to vignettes with closest match of general medical question types to Generic Question taxonomy [26] Click here for file

Additional file 4 **Information resource preferences identified in vignettes **Patient-specific and general medical information resources identified by participants in response to vignettes Click here for file

Additional file 5 **Frequency of perceived question types in vignettes **Patient-specific and general medical question types perceived by participants in response to vignettes according to frequency of report Click here for file

Additional file 6 **Frequency of identified information resource preferences in vignettes **Information resources identified by participants in response to vignettes according to frequency of report (for all vignettes and for vignettes by domain) Click here for file
