# Supplementary material for: Information resource preferences by general pediatricians in office settings: a qualitative study
Source: BMC Med Inform Decis Mak. 2005 Oct 14;5:34. doi: 10.1186/1472-6947-5-34 (PMC1266372; doi:10.1186/1472-6947-5-34)
Supplement: Additional file 1 — Vignettes used in semi-structured interviews Sixteen vignettes listed by domain (Genetics (8), Infectious Diseases(8)) [file 1472-6947-5-34-S1.doc]

# Additional file 1 – Vignettes used in semi-structured interviews

# Genetics

You are seeing a newborn infant for the first well-child visit. There are no problems noted, the child appears active, alert and on the 50th percentiles for growth. You note on physical examination Down facies and you hear a Grade III/VI systolic murmur. The mother mentions that she wasn’t sure, but she noticed that her baby’s face to have some blueness around the lips last night.

You are seeing a school-aged child who is visiting a family whom you normally follow. The child has sickle-cell disease and has been followed by a hematologist, but he hasn’t been there for “awhile” due to insurance issues. He has mild-to-moderate pain in the extremities, which he has had before, without fever or other constitutional symptoms or signs. He has a history of frequent hospitalizations with need for transfusions, but has not needed one for nearly a year.

You are seeing an elementary school-aged child as a new patient whose parents have noted the slow formation of bumps in the skin. The child is otherwise healthy and in no distress. Your examination reveals a number of firm, non-tender nodules on the arms and back, and pimple-like nodules on the face.

You are seeing a teenager recently diagnosed with cystic fibrosis. The parents are very concerned about the diagnosis as the child wants to become a professional athlete and they want to know about therapies that will cure the disease.

You are seeing a newborn infant who was born at home, delivered by an unlicensed nursing midwife. The infant is reported by the mother to have had no problems at delivery and has been breastfeeding well at home. On examination, the infant is active and alert, with slightly dysmorphic features, with what appears to be a repetitive, intermittent, unprovoked ‘startle’ reflex.

You are seeing a school-aged girl with a diagnosis of osteogenesis imperfecta from out of town. She states that she was running in the park when she heard a snap then had trouble walking and figured it was “time to see the doctor”. She appears to be in some discomfort and favors on leg. An x-ray shows a femoral fracture with other fractures of varying ages. The cast tech in the clinic puts her into a splint.

You are seeing a school-aged child whom you have been asked to evaluate for school failure. On examination, you note somewhat dysmorphic facies and a slight murmur on auscultation of the heart.

You are seeing a child who has been diagnosed with a metabolic defect for which there has been only palliative therapy. The parent has heard of other treatments that use gene therapy to alleviate and possibly cure the disease and wants to know more.

# Infectious diseases

You are seeing a 5 week old infant with a temperature of 100.4 F (37.9 C), rectally. It is winter and you have recently hospitalized two infants for possible sepsis and their cultures have all returned negative.

You are seeing a teenager for a sore throat and a cough. It is influenza season and there has been an outbreak of bacterial pneumonia within the population.

You are seeing a teenager referred right before school vacation for a “borderline” tuberculosis screening test that was performed in school. The school is in a very low-risk area, and you have been asked to make a recommendation on the school screening policy.

You are seeing a 10 year old male with sickle cell disease who was in an accident and developed osteomyelitis for which he received a course of intravenous antibiotic therapy. He is now at home and completing a course of oral therapy. He is doing well, and his antibacteriocidal titers are good. The parent asks how long will he have to be on therapy.

You are seeing a child with an acute onset of a rash you have never seen before. The child “looks ill.”

You are seeing a runaway teenager who recently became a ward of the state with a history of poly-substance abuse who is being followed in conjunction with a drug treatment program and is on a pharmacologic withdrawal protocol. He complains of extreme tiredness over the last 3 weeks. His vital signs are stable. His exam is significant for mild scleral icterus and slight right upper quadrant tenderness and easy bruising. His accompanying laboratory tests are notable for elevation of liver enzymes and abnormal coagulation functions.

You are seeing a runaway teenager who had been living on the streets to get away from an abusive home environment. On the streets, she was engaging in survival sex and began to use intravenous drugs. She was arrested after being admitted to the hospital for IV antibiotic treatment of cellulites of both arms. She is in custody and now presents to you (with her social worker and an officer) for health screening.

You are seeing a 4 week old infant born to a mother with a history of intravenous substance abuse who has been in a rehabilitation program since she discovered she was pregnant. During the course of her pregnancy, she discovered she was HIV positive and was started on ZDV monotherapy prior to the delivery of her child. The child is now in the clinic for evaluation.
