# Supplementary material for: Information resource preferences by general pediatricians in office settings: a qualitative study
Source: BMC Med Inform Decis Mak. 2005 Oct 14;5:34. doi: 10.1186/1472-6947-5-34 (PMC1266372; doi:10.1186/1472-6947-5-34)
Supplement: Additional file 2 — Information resource survey Relative frequency of resource use identified by participants. Cited uses of specific resources [file 1472-6947-5-34-S2.doc]

# Additional file 2 – Information resource survey

# Relative frequency of resource use identified by participants

| Frequently identified | Specialists, generalist colleagues, general pediatric texts  Formulary, specialty texts  Pediatric handbooks, professional organization Website  Guidelines, federal health Website  Electronic portals (when available), Abstracts |
| --- | --- |
| Infrequently identified | E-journals  E-texts  Medical librarian, Decision-making texts  Consumer health information  Evidence-based reviews |

# Cited uses of specific resources

| **Pediatric Handbook**  **(Harriet Lane Handbook)** | **Frequently cited uses:**   - **Drug dosing** - **Cardiology: EKG interpretation, BP/HR normals** - **Disease-specific protocols** |
| --- | --- |
|  | Other cited uses:   - Drug side-effects, general reference, nutrition, developmental information |
| **General texts**  **(Various)** | **Frequently cited uses:**   - **Review of specific diseases, infrequently seen and rare conditions** |
|  | Other cited uses:   - Decision support for diagnosis, testing and management - Clinical presentation of disease and photographic material |
| **Decision making aid**  **(Pediatric Decision Making)** | **Frequently cited uses:**   - **Not used** |
|  | Other cited uses:   - Differential diagnosis, rare or unfamiliar cases, teaching |
| **Formulary:**  **(Physician Desk Reference)** | **Frequently cited uses:**   - **Drugs: dosage, adverse reactions, available forms** |
|  | Other cited uses:   - Picture identification, interactions, unfamiliar and new drugs, indications/contraindications, OTC information, not used |
| **Specialty texts**  **(Various)** | **Frequently cited uses:**   - **Infectious disease/vaccines** - **Genetics** - **Dermatology** |
|  | Other cited uses:   - Musculoskeletal problems, cardiology, child abuse, non-specifie problems, Don’t use - Recommendations, Test interpretation, Differential diagnosis, Drug dosage/interactions, Breastfeeding information, Teaching |
| **Evidence-based reviews (Cochrane Collection)** | **Frequently cited uses:**   - **Don't Use** |
|  | Other cited uses:   - Education, Best treatment, general management |
| **Guidelines:** | Cited uses:   - Problem management - Reading/self-education, asking general questions - Reference for publication, policy writing - Not used |
| **Abstracts**  **(PubMed)** | Cited uses:   - Not used in office - For continuing education, To answer questions - To learn about new research, To get a specific citation, For use if no policy exists, For teaching, For debates |
| **Professional colleague (generalist)** | **Frequently cited uses:**   - **Sounding board (to confirm diagnosis, examination or management)** |
|  | Other cited uses:   - Diagnostic dilemmas, Local epidemiology |
|  | Method of contact   - Face-to-face, Telephone |
| **Specialist:** | **Frequently cited uses:**   - **Referral: diagnosed problem, procedure, care beyond one’s own capability** |
|  | Other cited uses:   - Advice: Pre-referral workup, management plan, diagnostic dilemma, patient-specific questions (established relationship) |
|  | Method of contact:   - Telephone through established referral network |
| Medical Librarian | **Frequently cited uses:**   - **Not used (Reasons: "Lack of proximity”, “Not used since training")** |
|  | Other cited reasons:   - Reference for writing, To answer specific question, Self-education and teaching |
| Consumer health information **(Medem)** | **Frequently cited uses:**   - **Not used (except when provided by work environment)** |
|  | Other cited uses:   - For patient education (on practice Website), Self-education on what parents read |
| **Professional association site (AAP):** | **Frequently cited uses:**   - **Policies and guidelines** |
|  | Other cited uses:   - Patient education, Meeting information, Professional education, Journals, Travel information (professional), Professional news, ADHD questionnaires, Advocacy - Not used |
| **Federal health Website**  **(NIH, CDC, FDA)** | **Frequently cited uses:**   - **Travel health, Immunizations** - **Most commonly cited Website: CDC Travel Health** |
|  | Other cited uses:   - Infectious diseases (STDs, Bioterrorism, Public Health), Obesity, News, Drug information - Not used |
| **Online journals:** | **Frequently cited reasons:**   - **Not used in office (preference for paper versions)** |
|  | Other cited uses:   - For specific articles of interest, General reading and keeping up, Overview of a problem |
|  | Specific journals mentioned:   - Contemporary Pediatrics, Pediatrics, Journal of Pediatrics, American Journal of Public Health, MDConsult, Medscape |
| **Online medical texts:** | **Frequently cited uses:**   - **Not used (Reasons: preference for print, cost as barrier)** |
|  | Other cited uses:   - Similar to print texts, searching multiple texts |
|  | Specific texts mentioned:   - Nelson’s (General text), Harrison’s (General medical), Mandell (Infectious disease), AAP Red Book (Pediatric infectious disease) |
| **Online portal** | **Frequently cited uses:**   - **Not used or used rarely** |
|  | Other cited uses   - CME tracking, Research, Guidelines, Patient education (English and Spanish) |
|  | Online resources mentioned:   - PediaLink, MDConsult, Medscape |
| Other resources | Resources mentioned   - Up-To-Date, Micromedex (Commercial portals) - Dermatology, allergy, breastfeeding, gynecology texts, Formularies AAP Policy Books, AAP Patient education - Testing materials: PREP - Online databases: OMIM (Genetics), MEDLINE (Abstracts), Dermatlas (Dermatology) - Online communication: PedTalk (mailing list), PedsChat (professional chatroom) - PDA: ePocrates (formulary), medical texts - Websites: Local health departments, Google - Pharmacy-sponsored conferences - Lay press |
