# Supplementary material for: Information resource preferences by general pediatricians in office settings: a qualitative study
Source: BMC Med Inform Decis Mak. 2005 Oct 14;5:34. doi: 10.1186/1472-6947-5-34 (PMC1266372; doi:10.1186/1472-6947-5-34)
Supplement: Additional file 3 — Question types perceived in vignettes Patient-specific and general medical question types perceived by participants in response to vignettes with closest match of general medical question types to Generic Question taxonomy [26] [file 1472-6947-5-34-S3.doc]

# Additional file 3 – Question types perceived in vignettes

1 Patient-specific

1.1 Demographics (What is pt's age?)

1.2 Administrative

1.2.1 Legal status (Who is pt's guardian?)

1.2.2 Insurance (What is pt's financial status?)

1.3 History

1.3.1 Past diagnosis/examination (What have been past dx/findings?)

1.3.2 Exposures (What have been exposures?)

1.3.3 Treatments (What have been past tx?)

1.3.3.1 Duration (How long/frequent has problem been?)

- - - 1. Course/Response (What has pt's course/response with dx

been?)

1.3.4 Risk (What is pt's genetic, social risk?)

1.3.5 Social history (What is pt's SH?)

1.4 Findings

1.4.1 Symptom (Questions about sx)

1.4.2 Sign (Questions about physical)

1.4.3 Test result (Questions about pt's test results)

1.4.4 Etiology (Questions about cause of problem in this pt?)

1.5 Assessment/status

1.5.1 Clinical stability (Is pt clinically stable?)

1.5.2 Emotional stability (Is pt emotionally stable?)

1.5.3 Appearance (How does pt seem?)

1.5.4 Cognitive (What are pt's cog issues: lit/num/health

lit/undrstd?)

1.6 Diagnosis

1.6.1 Disease or condition

1.6.1.1 Etiology (What caused this in this pt?)

1.6.1.2 Findings/manifestation (What pt findings are

associated with this?)

1.6.1.3 Risk (What is the pt's risk?)

1.6.1.4 Severity (What is the severity of the problem?)

1.6.1.5 Clinical course (What has the pt's RECENT

course with the disease been?)

1.6.1.6 Acuity (How acute is it?)

1.7 Treatment

1.7.1 Current (What is the pt's current tx?)

1.7.2 Plan (What is the plan for tx?)

1.7.3 Schedule follow-up/referral (When, where is pt's schedule,

next visit?)

1.7.4 Choices/types (What are the choices for the pt?)

1.7.5 Cognitive (What are appropriate cognitive resources for pt?)

1.7.6 Socioeconomic issues (What are SES issues?)

1.7.7 Compliance (What is pt's compliance?)

2 General medical [Matches to Generic Question Taxonomy (Ref 26)]

2.1 Diagnosis, disease, syndrome, condition

2.1.1 Background information

2.1.1.1 Classification (What are the forms of this dx?) [No match]

2.1.1.2 Pathophysiology (What is the mechanism of

this dx?) [8.3.1.1]

2.2.2 Manifestation/findings

2.2.2.1 Signs/symptoms (What are sx/signs of dx?) [2.1.1/2.1]

2.2.2.2 Tests (What are tests to run for dx?) [2.2.1.1]

2.2.3 Consequence of delay (Can I wait to dx?) [4.2.1.1]

2.2 Treatment

2.2.1 Current (What is state-of-the-art tx?) [3.1.10.1]

2.2.2 Best (What is 'best' tx?) [3.1.10.1]

2.2.3 Available (What is available tx?) [No Match]

2.2.4 Counseling (Any generic counseling) [4.5.1.1]

2.2.5 Consequence of delay (Can I wait to tx?) [4.2.1.1]

2.2.6 Criteria for (What are dx criteria to tx?) [3.1.6.1]

- - 1. Effects/side effects of (What are effects/side-effects of tx?)

[3.1.5.x]

2.2.8 Cost of (What is cost of tx?) [3.1.12.3]

2.2.9 Protocols/steps (What are steps of tx?) [3.2.1.1]

3 Other (specify)
