# Supplementary material for: Information resource preferences by general pediatricians in office settings: a qualitative study
Source: BMC Med Inform Decis Mak. 2005 Oct 14;5:34. doi: 10.1186/1472-6947-5-34 (PMC1266372; doi:10.1186/1472-6947-5-34)
Supplement: Additional file 4 — Information resource preferences identified in vignettes Patient-specific and general medical information resources identified by participants in response to vignettes [file 1472-6947-5-34-S4.doc]

# Additional file 4 – Information resource preferences identified in vignettes

1 Patient-specific information resources

1.1 Patient or guardian

1.1.1 Any history

1.1.2 Any examination

1.1.3 Any Sign or Symptom

1.2 Provider-based information about patient

1.2.1 Medical record

1.2.1.1 Previous provider

1.2.1.1.1 Discharge summary

1.2.1.1.2 Hospital

1.2.1.2 Concomitant provider

1.2.2 Test result

1.2.3 Current provider (Self)

1.2.3.1 Training

1.2.3.2 Experience

2 General medical information resources

2.1 Persons

2.1.1 Colleague (generalist)

2.1.2 Specialist (cardiologist, neurologist, etc.)

2.1.3 Librarian (search intermediary)

2.2 Printed sources

2.2.1 Textbooks

2.2.1.1 General pediatric text

2.2.1.2 Specialty pediatric text

2.2.2 References

2.2.2.1 Handbook

2.2.2.2 Formulary

2.2.2.3 Decision-support aid

2.2.3 Journals

2.2.3.1 Professional journals

2.2.3.2 Evidence-based reviews

2.2.3.3 Consumer health information – Lay press

2.3 Electronic resources

2.3.1 Electronic “print” sources (e-versions of 2.2)

2.3.1.1 Medical portals (collections of sources)

2.3.1.2 Online/PDA texts and handbooks

2.3.1.2.1 Formulary

2.3.3 Websites

2.3.3.1 Professional organization

2.3.3.1.1 Guidelines

2.3.3.1.2 Consumer health information

2.3.3.1.3 Discussion groups

2.3.3.2 Government health information

2.3.2.2.1 Federal health information sources

2.3.2.2.2 Local health information sources

(ie immunization registry)

2.3.4 Research

2.3.4.1 Evidence-based reviews

2.3.4.2 Abstracts or research articles

2.3.5 Practice information

2.3.5.1 Practice database

3 Other
