# Supplementary material for: Information resource preferences by general pediatricians in office settings: a qualitative study
Source: BMC Med Inform Decis Mak. 2005 Oct 14;5:34. doi: 10.1186/1472-6947-5-34 (PMC1266372; doi:10.1186/1472-6947-5-34)
Supplement: Additional file 5 — Frequency of perceived question types in vignettes Patient-specific and general medical question types perceived by participants in response to vignettes according to frequency of report [file 1472-6947-5-34-S5.doc]

**Additional file 5 – Frequency of perceived question types in vignettes**

All (patient-specific (PS) and general medical (GM)) question types

| **Question Type (PS = Patient-specifc; GM = General Medical)** | **No.** | **% of all perceived questions** |
| --- | --- | --- |
| PS-What is the patient's diagnosis? | 38 | 12.22% |
| PS-What is the result of the exam, test? | 34 | 10.93% |
| PS-What was the previous management? | 23 | 7.40% |
| PS-What is the present history? | 18 | 5.79% |
| PS-What is the past history? | 14 | 4.50% |
| PS-What is the current management? | 13 | 4.18% |
| GM-What are diagnostic guidelines for a disease? | 12 | 3.86% |
| GM-What are treatment guidelines for a disease? | 12 | 3.86% |
| GM-What is the etiology of the problem? | 11 | 3.54% |
| PS-What is the patient's risk? | 10 | 3.22% |
| PS-What are the patient's exposures? | 9 | 2.89% |
| PS-What are the patient's medications? | 9 | 2.89% |
| PS-What is the patient social history, network? | 9 | 2.89% |
| PS-Is the patient stable? | 8 | 2.57% |
| PS-What is the patient or family's understanding of a disease? | 8 | 2.57% |
| PS-How severe is the problem? | 7 | 2.25% |
| PS-What has been the patient's clinical course? | 7 | 2.25% |
| GM-What is the disease classification? | 6 | 1.93% |

Only general medical question types

| **General Medical Question Type** | **No.** | **% of all perceived general medical questions** |
| --- | --- | --- |
| What are diagnostic guidelines for a disease? | 12 | 18.46% |
| What are treatment guidelines for a disease? | 12 | 18.46% |
| What is the etiology of the problem? | 11 | 16.92% |
| What is the disease classification? | 6 | 9.23% |
| What are referral criteria for a disease? | 5 | 7.69% |
| What are sources of patient information on a disease? | 5 | 7.69% |
| What is the latest/best the treatment of a disease? | 4 | 6.15% |
| What findings are associated with this disease? | 3 | 4.62% |
| How is a test performed properly? | 2 | 3.08% |
| How is a test interpreted? | 1 | 1.54% |
| What is the cost of a test to diagnose a disease? | 1 | 1.54% |
| What is the effect of a drug on a disease? | 1 | 1.54% |
| What is the prevalence of a disease? | 1 | 1.54% |
| Will insurance cover a treatment for a disease? | 1 | 1.54% |
