# Supplementary material for: Information resource preferences by general pediatricians in office settings: a qualitative study
Source: BMC Med Inform Decis Mak. 2005 Oct 14;5:34. doi: 10.1186/1472-6947-5-34 (PMC1266372; doi:10.1186/1472-6947-5-34)
Supplement: Additional file 6 — Frequency of identified information resource preferences in vignettes Information resources identified by participants in response to vignettes according to frequency of report (for all vignettes and for vignettes by domain) [file 1472-6947-5-34-S6.doc]

**Additional file 6 – Frequency of identified information resource preferences in vignettes**

All vignettes

| Information resource | No. | % of all identified resources |
| --- | --- | --- |
| Specialist | 73 | 25.3 |
| Textbook-Specialty | 25 | 8.7 |
| Patient-History | 22 | 7.6 |
| Patient-Exam, Test Results | 21 | 7.3 |
| Generalist | 16 | 5.6 |
| Textbook-General Pediatrics | 13 | 4.5 |
| Online-General Search Engine | 12 | 4.2 |
| Online-Portal | 10 | 3.5 |
| Self knowledge | 10 | 3.5 |
| Institution-Hospital | 9 | 3.1 |
| Record | 9 | 3.1 |
| Guidelines-Professional | 7 | 2.4 |
| Institution-Social Services | 6 | 2.1 |
| Online-Database | 6 | 2.1 |
| Government-Local health department | 5 | 1.7 |
| Institution-School | 5 | 1.7 |

Vignettes by domain (*percent of all identified resources by domain)

| **Genetics** | **No** | **%*** | **Infectious diseases** | **No.** | **%*** |
| --- | --- | --- | --- | --- | --- |
| History from family, caretakers or guardians | 12 | 8.70 | Laboratory test results | 12 | 7.95 |
| Specialist-genetics | 9 | 6.52 | History from family, caretakers or guardians | 11 | 7.28 |
| Textbook-General Pediatrics | 9 | 6.52 | General pediatrician | 9 | 5.96 |
| Web-SearchEngine-Google | 8 | 5.80 | Specialist-Infectious disease | 9 | 5.96 |
| General pediatrician | 7 | 5.07 | No particular resource. Own knowledge. | 7 | 4.64 |
| Specialist-Cardiologist | 7 | 5.07 | Guidelines from the AAP | 6 | 3.97 |
| Laboratory test results | 6 | 4.35 | Hospital | 6 | 3.97 |
| Online specialty information portal | 6 | 4.35 | Government-Local health department | 5 | 3.31 |
| Specialty text: dysmorphology atlas | 6 | 4.35 | Medical record | 5 | 3.31 |
| Online database – OMIM | 5 | 3.62 | Social services | 5 | 3.31 |
| Medical record | 4 | 2.90 | Specialist-Hematology-Oncologist | 5 | 3.31 |
| Specialist-not specified | 4 | 2.90 | Specialist-HIV | 5 | 3.31 |
| Specialist-orthopedist | 4 | 2.90 | Government-CDC-Website | 4 | 2.65 |
| Specialist-Pulmonologist | 4 | 2.90 | Online specialty information portal | 4 | 2.65 |
| Specialty textbook-dermatology | 4 | 2.90 | Specialty text Infectious Disease | 4 | 2.65 |
| Hospital | 3 | 2.17 | Specialty textbook-Infectious disease | 4 | 2.65 |
| No particular resource. Own knowledge. | 3 | 2.17 | Textbook-General Pediatrics | 4 | 2.65 |
| Online abstracts-PubMed | 3 | 2.17 | Web-SearchEngine-Google | 4 | 2.65 |
| Patient's school | 3 | 2.17 | Specialist-dermatologist | 3 | 1.99 |
| Specialist-neurologist | 3 | 2.17 | Specialist-Gastroenterologist | 3 | 1.99 |
| Decision support - Clinical consult - PDA | 2 | 1.45 | Specialist-Psychiatry | 3 | 1.99 |
| Health education for patients, parents | 2 | 1.45 | Specialty textbook-dermatology | 3 | 1.99 |
